# Cyclic Dipeptides Formation From Linear Dipeptides Under Potentially Prebiotic Earth Conditions

**DOI:** 10.3389/fchem.2021.675821

**Published:** 2021-06-28

**Authors:** Yeting Guo, Jianxi Ying, Dongru Sun, Yumeng Zhang, Minyang Zheng, Ruiwen Ding, Yan Liu, Yufen Zhao

**Affiliations:** ^1^ Institute of Drug Discovery Technology, Ningbo University, Ningbo, China; ^2^ Qian Xuesen Collaborative Research Center of Astrochemistry and Space Life Sciences, Ningbo University, Ningbo, China; ^3^ Department of Chemical Biology, Key Laboratory for Chemical Biology of Fujian Province, College of Chemistry and Chemical Engineering, Xiamen University, Xiamen, China; ^4^ Key Laboratory of Bioorganic Phosphorus Chemistry and Chemical Biology, Ministry of Education, Department of Chemistry, Tsinghua University, Beijing, China

**Keywords:** origin of life, prebiotic synthesis, cyclic dipeptide, linear dipeptide, aqueous solution

## Abstract

Cyclic dipeptides (DKPs) are peptide precursors and chiral catalysts in the prebiotic process. This study reports proline-containing DKPs that were spontaneously obtained from linear dipeptides under an aqueous solution. Significantly, the yields of DKPs were affected by the sequence of linear dipeptides and whether the reaction contains trimetaphosphate. These findings provide the possibility that DKPs might play a key role in the origin of life.

## Introduction

The biosynthesis of peptides and the chirality of biomolecules are induced in cells by the catalysis of enzymes. Homochirality is an essential feature of life (Bonner, 2000). The enzymatic processes are extremely precise and complex and cannot be realized at the beginning of life. For the origin of life, simpler forms must have driven the prebiotic evolution process (Ying et al., 2018). The prebiotic peptide synthesis and the origin of bio-chirality remain an abstruse problem. Cyclic dipeptides [2, 5-diketopiperazines, (DKPs)] have been found to have peptides elongating and chiral catalysis properties (Mauger, 1968; Bycroft, 1969; Poullennec et al., 2002; Huber et al., 2003; Borthwick, 2012; Danger et al., 2012; Petsi and Zografos, 2020). The DKPs could act as activated intermediates in the formation of peptides as was suggested by Yamagata *et al.* and Imai *et al* (Nagayama et al., 1990; Takaoka et al., 1991; Imai et al., 1999). The DKPs might exert the chiral catalytic function prior to the appearance of the enzyme in the chemical evolution process. Thus, it is reasonable to believe that the DKPs could have played an essential role in the origin of life, and because of this, it is worth exploring whether the DKPs could be formed in prebiotic conditions.

A number of experiments have been implemented concerning how cyclic dipeptides were formed, but there is limited research involving the formation of cyclic dipeptides in an aqueous solution with prebiotically plausible conditions (Botti et al., 1996; Trauger et al., 2000; Martins and Carvalho, 2007; Jainta et al., 2008; Nonappa et al., 2011). We recently found that an alkaline aqueous solution with trimetaphosphate (P_3_m) drives proline-containing DKPs formation starting from free amino acids (Ying et al., 2018). Experimental studies have shown that P_3_m has similar abilities to the polymerization of biomolecules such as amino acids and nucleotides (Rabinowitz et al., 1969; Gao and Orgel, 2000; Gibard et al., 2018; Sibilska et al., 2018; Ying et al., 2019). Yamagata *et al.* demonstrated that volcanic activities produced water-soluble P_3_m (Yamagata et al., 1991). Since most of the phosphates are insoluble apatites, P_3_m may be essential for the prebiotic chemical evolution process.

In our assays, proline-containing DKPs (cyclo-Pro-AAs) with species of up to 12 amino acid residues were detected (Ying et al., 2018). A possible mechanism for the formation of cyclo-Pro-AAs has been deduced, which may undergo two steps, the formation and then the cyclization of linear dipeptide. How the effect of P_3_m and the sequence of linear dipeptides on the formation of cyclo-Pro-AAs in simulated prebiotic conditions has been unclear. Thus far, only linear dipeptide Pro-Pro (linear-Pro-Pro) spontaneous forming cyclo-Pro-Pro had been tested and it was unclear whether other proline-containing dipeptide sequences (linear-Pro-AA and linear-AA-Pro) have an effect on the formation of the corresponding cyclo-Pro-AA, and whether P_3_m affects its cyclization process. In this paper, we experimentally investigated the cyclization of proline-containing dipeptides from the above two aspects.

## Materials and Methods

### Materials

Prolylglycine (linear-Pro-Gly), Glycylproline (linear-Gly-Pro), Prolylalanine (linear-Pro-Ala), Alanylproline (linear-Ala-Pro), Valylproline (linear-Val-Pro), Histidylproline (linear-His-Pro), and Alanylglycylproline (linear-Ala-Gly-Pro) were obtained from Anhui Guoping Pharmaceutical Co. LTD. Trimetaphosphate and Formic acid were purchased from Sigma Aldrich. Methanol (HPLC grade) was purchased from SpectrumChemical. Ultrapure water (18.2 MΩ cm) from a Milli-Q water purification system (Millipore, Bedford, MA) was used to prepare solutions and the mobile phase. Reagents were obtained and verified by MS spectra (Supplementary Figures S1–S7).

### General Procedure for Synthesizing Cyclic Dipeptides

0.1 mmol linear dipeptide (linear-Pro-Gly, linear-Gly-Pro, linear-Pro-Ala, linear-Ala-Pro, linear-Val-Pro, and linear-His-Pro) was mixed with or without 0.1 mmol sodium trimetaphosphate (P_3_m) in 1 mL alkaline aqueous solution, respectively. The pH of the reaction mixture was adjusted to 11 using 10 M NaOH. Then the reaction mixtures were placed at 55°C for 1 day. The reaction was quenched with 6 M HCl solution.

### General Procedure for Synthesizing Cyclic Tripeptide

0.1 mmol sodium trimetaphosphate (P_3_m) was mixed with 0.1 mmol linear-Ala-Gly-Pro in a 1 mL alkaline aqueous solution. The pH of the reaction mixture was adjusted to 11 using 10 M NaOH. Then the reaction mixture was placed at 55°C for 1 day. The reaction was quenched with 6 M HCl solution.

### Analysis Methods

#### MS

For reagent linear dipeptides and tripeptides, MS and MS^2^ were performed on the Q-Exactive Plus system in positive mode. The MS instrument parameters were as follows: spray voltage of 3800 V, sheath gas flow rate of 3 L•min^−1^, the capillary temperature at 320°C. Mass spectra were registered in the scan range from m/z = 100 to 600.

#### HPLC-MS

MS was performed on a Bruker micrOTOF-Q Ⅱ system in positive mode. The MS instrument parameters were as follows: capillary voltage of 4500 V, nebulizer pressure of 2 bar, dry gas of 8 L•min^−1^, and dry temperature at 200°C. Mass spectra were registered in the scan range from *m/z* = 50 to 1,000. For ESI-MS, about 1/10 of the eluate from LC was introduced through a splitting T valve. As for the on-line detection of the reaction product by HPLC-MS, we set up the divert valve of the MS instrument as follows: 1) when the divert valve was in waste position, the valve can be used for switching HPLC flows directly to waste for about 3 min; 2) after that, when the divert valve was in source position, the valve can be used for switching HPLC flows directly to MS.

The HPLC was performed on Agilent 1,260 Infinity system and fitted with an Agilent TC-C18, 5 µm, 4.6 mm × 150 mm column. The column temperature was maintained at room temperature. A binary mobile phase (solvent A: water with 0.01% formic acid; solvent B: methanol) was used with the flow rate at 0.8 mL•min^−1^. The linear gradient elution program was as follows: 0∼15 min, 5∼30% B; 15∼20 min, 30%–70% B; 20∼23 min, 70% B; 23∼25 min, 70∼5% B; 25∼30 min, 5% B.

## Results and Discussion

We initially investigated whether the sequence of dipeptides would affect the formation of cyclo-Pro-AAs. We chose linear dipeptide prolylglycine (linear-Pro-Gly) and glycylproline (linear-Gly-Pro) as the model substrate to react in an aqueous solution of pH 11 at 55°C for 1 day, respectively. The pH was not adjusted during the whole process. All the experimental data reported here were obtained after repeating the experiments three times.

**FIGURE 1:**
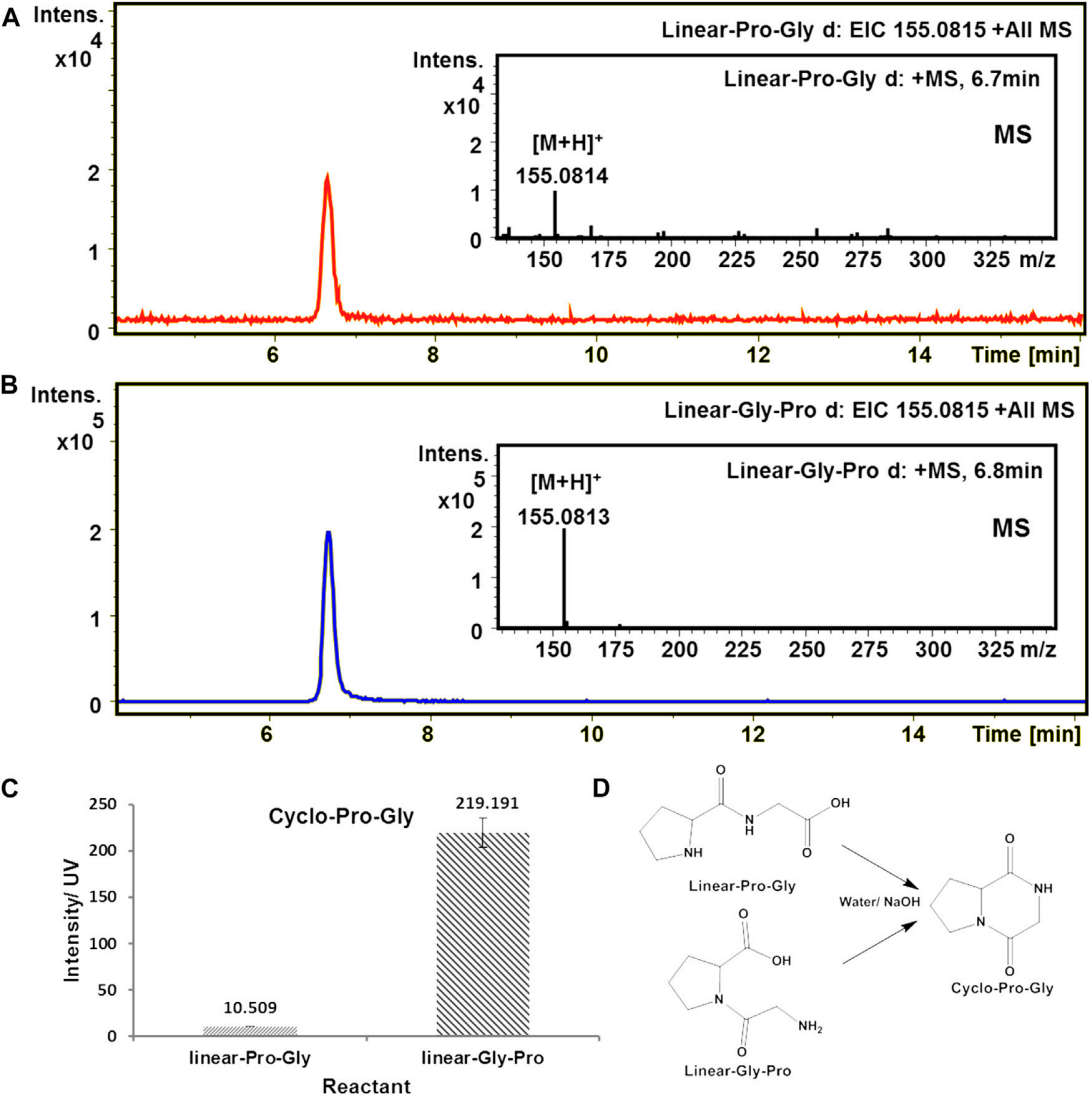
Formation of cyclo-Pro-Gly from the cyclization of the linear dipeptide in an alkaline aqueous solution (pH = 11). The calculated value of cyclo-Pro-Gly [M + H]^+^ is 155.0815. **(A)** EIC-MS profile of the product cyclo-Pro-Gly from the cyclization of linear-Pro-Gly. **(B)** EIC-MS profile of the product cyclo-Pro-Gly from the cyclization of linear-Gly-Pro. **(C)** The amount of cyclo-Pro-Gly was produced from linear-Pro-Gly and linear-Gly-Pro, respectively. **(D)** The reaction of the cyclo-Pro-Gly formation through the cyclization of linear-Pro-Gly and linear-Gly-Pro, respectively.

Using HPLC-high resolution mass spectrometry (HPLC-HRMS) for sample analysis, the extracted ion chromatogram (EIC) of m/z 155.0815 gives one peak with a retention time of 6.7 min, which corresponds to cyclo-Pro-Gly molecular ion [M + H]^+^. These results indicated that both linear-Pro-Gly and linear-Gly-Pro could form cyclo-Pro-Gly under alkaline aqueous solution. Remarkably, the yield of cyclo-Pro-Gly from linear-Gly-Pro was higher than that from linear-Pro-Gly, which was improved up to 20.9 times (Figure 1 and Supplementary Table S1).

DFT calculations were performed with the Gaussian 16 suite of quantum chemical packages. The spin-unrestricted B3LYP functional was employed with a 6-31G** basis set for all atoms. Transition states were ascertained by vibrational frequency analysis to possess a single mode along the reaction path with only one imaginary frequency. All optimizations and single point calculations were performed with solvation included using the self-consistent reaction field (SCRF) calculations, in the conductor-like polarizable continuum model (CPCM). The experimental solvent water was used. An experimental temperature of 293.15K was adopted in the calculations of Gibbs free energy. Our modeling results indicate that the cyclization of linear dipeptides has a negative standard free enthalpy of reaction (Supplementary Table S2), and thus should be favored in solution. However, the activation energy may be high, and therefore cyclic dipeptide formation may be too slow in the experiments with P_3_m. This would suggest that the promoting effect of P_3_m on cyclization is kinetic rather than thermodynamic.

**FIGURE 2:**
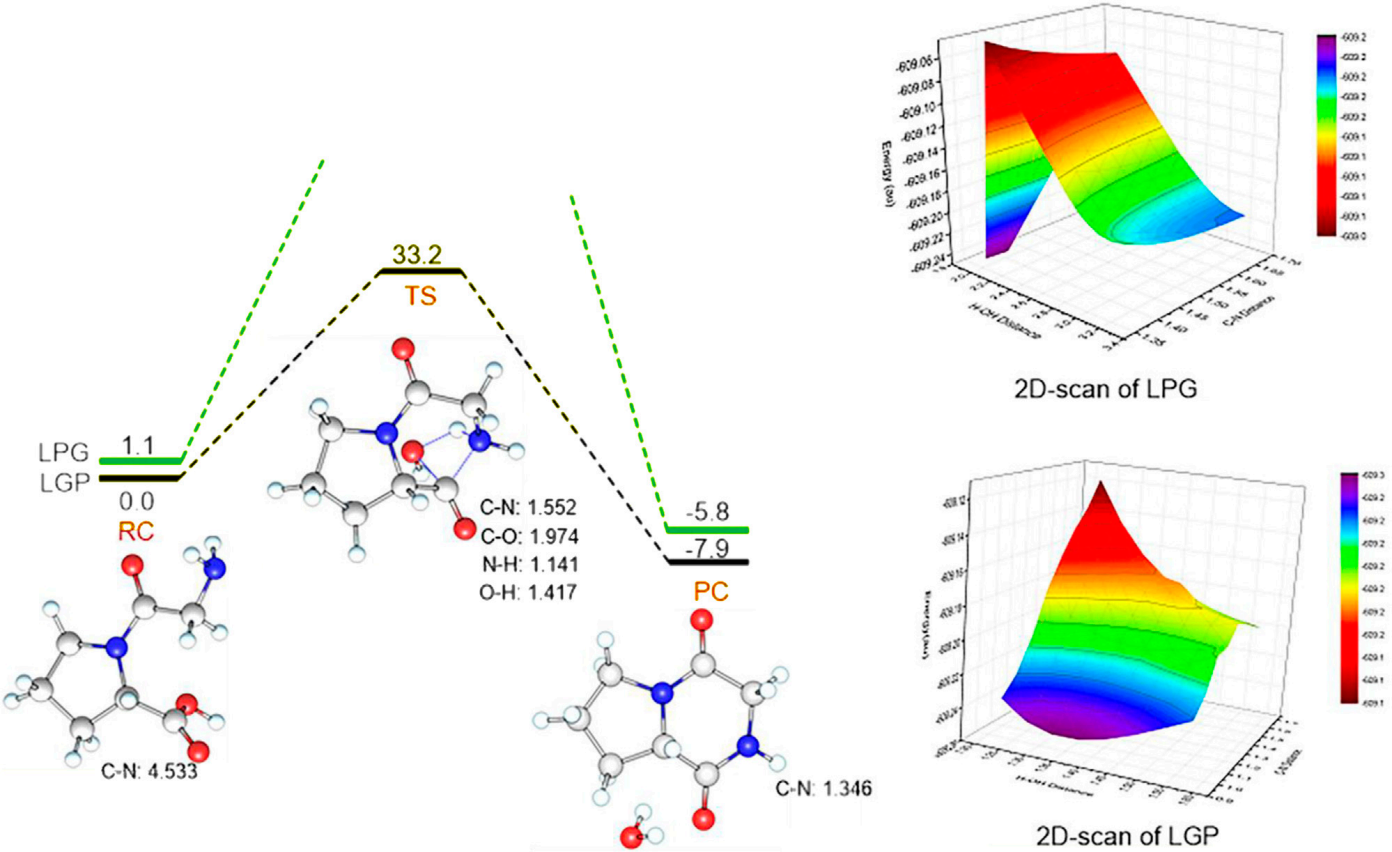
Energy profiles (in kcal·mol^−1^) for the Linear-Gly-Pro and Linear-Pro-Gly to form cyclo-Pro-Gly. Key geometric information on transition states is presented. Energies are in kcal mol^−1^ units, lengths are in Å units, angles are in degree units. LPG: Linear-Pro-Gly; LGP: Linear-Gly-Pro.

These theoretical calculations were consistent with the experimental results above. Linear-Gly-Pro is easier to form cyclic compounds than linear-Pro-Gly with a lower barrier of 33.2 kcal mol^−1^. For the transition state, the C-N distance is 1.552 Å, C-O one is 1.974 Å, the N-H one is 1.141 Å, and O-H one is 1.417 Å. The products are cyclic dipeptide. This step is an exothermic process with large heat (41.1 kcal·mol^−1^) (Figure 2). In the case of linear-Pro-Gly, the energy continued to rise during the scanning process and no transition state was found. Meanwhile, it can also be found from Figure 2 that the 2D-scan of linear-Pro-Gly (LPG) had no saddle points. The energy line between the reactants and the products showed that the energy of linear-Pro-Gly was higher than the energy of linear-Gly-Pro. This qualitative analysis was therefore meaningful for the case. Furthermore, the products of cyclization of linear-Pro-Gly and linear-Gly-Pro were the same, namely cyclo-Pro-Gly. Although the products were the same, they had different binding positions with the water produced by the reactions, respectively. Therefore, the atoms involved in the formation of hydrogen bonds were different, which leads to a certain deviation in the calculated energy levels, −5.8 and −7.9 kcal/mol (Figure 2). For the reactions, we preferred to focus on comparing the energy gap of the transition states with the experiment results, so we did not discuss the products in this section.

Similar reactions with linear dipeptide prolylalanine (linear-Pro-Ala) and alanylproline (linear-Ala-Pro) have led to the formation of cyclo-Pro-Ala, respectively. Similar to the above reactions, the experiment containing linear-Ala-Pro produced more cyclo-Pro-Ala than that containing linear-Pro-Ala by a factor of 7.5 (Supplementary Figure S8). These results showed that the experiment containing linear-AA-Pro produced more cyclo-Pro-AA than that containing linear-Pro-AA.

**FIGURE 3:**
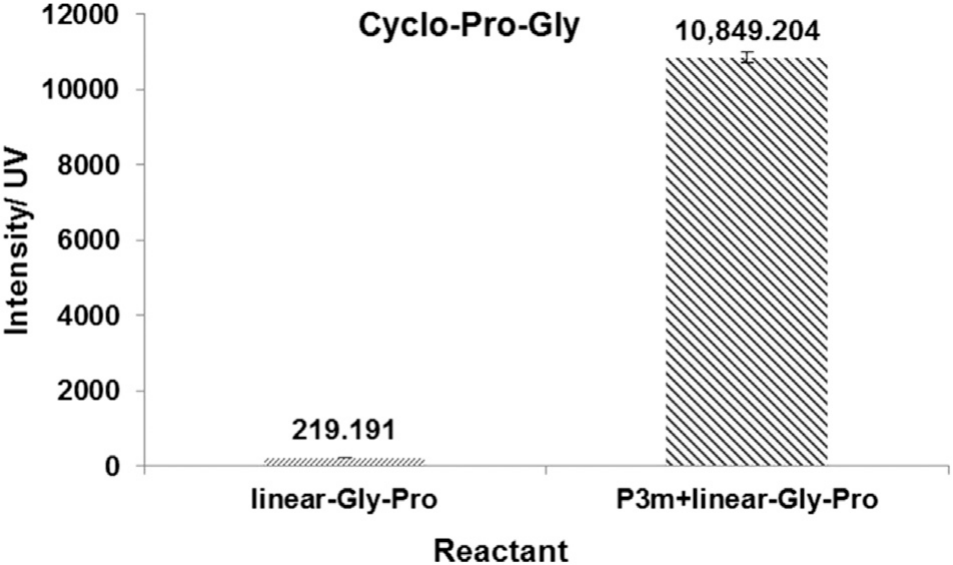
Effects of P_3_m on the formation of cyclo-Pro-Gly in the reaction of linear-Gly-Pro with or without the presence of P_3_m. All reactions were controlled under standard conditions.

Trimetaphosphate-induced linear peptide synthesis, amino acids in aqueous solutions, have been widely known for several decades (Rabinowitz et al., 1969; Hill and Orgel, 2002; Sibilska et al., 2017). We aimed to discover whether P_3_m could promote the formation of cyclic dipeptides from cyclization of linear dipeptides under potentially prebiotic aqueous conditions. For this purpose, we then studied the reaction of P_3_m with four proline-containing linear dipeptides (linear-Pro-Gly, linear-Gly-Pro, linear-Pro-Ala, and linear-Ala-Pro), respectively. The corresponding cyclic dipeptides were all detected from the resulting solutions by HPLC-HRMS (Supplementary Figure S9 and Supplementary Table S1). The yields of cyclo-Pro-AA of the reaction system containing P_3_m were much higher than those without P_3_m. Their yields are shown in Figure 3 and Supplementary Figure S10.

These results showed that the yield of cyclo-Pro-AAs could be significantly increased by P_3_m. It is worth noting that P_3_m was unstable and underwent hydrolysis to give pyrophosphate (PPi) and phosphate (Pi) in the alkaline aqueous solution. Therefore, we then wondered which salt (P_3_m, PPi, or Pi) was responsible for promoting the formation of cyclic dipeptides. To solve this puzzle, reaction mixtures containing varied species of phosphates (PPi and Pi) and corresponding linear dipeptides (linear-Pro-Gly, linear-Gly-Pro, linear-Pro-Ala, and linear-Ala-Pro) were incubated, respectively, at 55°C in an aqueous solution with the pH 11 for 1 day. Pi never has any promoting effect on ring closure–if anything, it exhibited a negative effect. Indeed, monomeric phosphate is not a high-free energy molecule and should not be able to have a promoting thermodynamic effect on amide bond formation. As for PPi, which contains one high-free energy P-O-P bond, it did have a positive effect in one case, namely when starting from linear-Pro-Gly (Supplementary Figures S11–S13). PPi and Pi were not considered in later experiments. It was therefore implied that P_3_m had a positive effect on cyclo-Pro-AAs formation.

**FIGURE 4:**
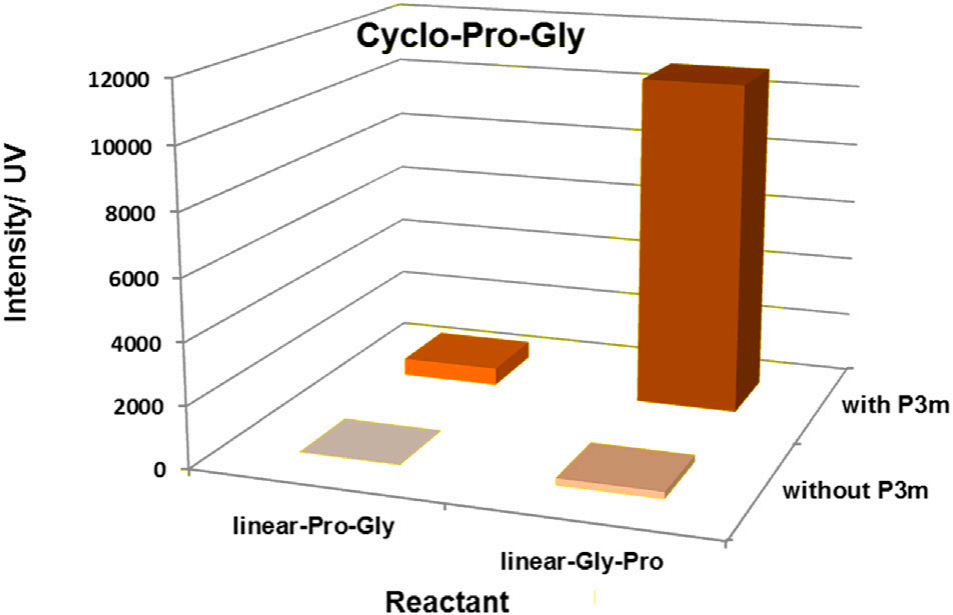
Effect of P_3_m and the sequence of linear dipeptides on the formation of cyclo-Pro-Gly. All reactions were controlled under standard conditions.

Based on the above experiments, the results showed that the yield of cyclo-Pro-AAs was affected by the sequence of linear dipeptides and whether the reaction system contains P_3_m. That is to say, the yield of cyclo-Pro-AA from linear-AA-Pro as reactant was increased compared with that from linear-Pro-AA as the reactant. The yield of the cyclo-Pro-AA could also be promoted by the presence of P_3_m in the reaction (Figure 4; Table 1, and Supplementary Figure S14).

**TABLE 1:** Relative abundance of the products from the reaction of proline-containing linear dipeptides with or without P_3_m.

Entry	Reactant		Product	Relative abundance (%)^a^
	P_3_m	Linear dipeptide		
1	+^b^	Linear-Ala-Pro	Cyclo-Pro-Ala	100
2	−^c^	Linear-Ala-Pro		13.7
3	+^b^	Linear-Pro-Ala		6.2
4	−^c^	Linear-Pro-Ala		1.8
5	+^b^	Linear-Gly-Pro	Cyclo-Pro-Gly	100
6	−^c^	Linear-Gly-Pro		2.0
7	+^b^	Linear-Pro-Gly		5.3
8	−^c^	Linear-Pro-Gly		0.1
9	+^b^	Linear-Val-Pro	Cyclo-Pro-Val	100
10	−^c^	Linear-Val-Pro		10.2
11	+^b^	Linear-His-Pro	Cyclo-Pro-His	100
12	−^c^	Linear-His-Pro		9.7

a The relative abundance was calculated by the integral area at 210 nm.

b The reaction of proline-containing linear dipeptide with P_3_m.

c The reaction of proline-containing linear dipeptide without P_3_m.

We then investigated whether other proline-containing linear dipeptides which have more complex side chains of another amino acid besides proline, showed similar behavior to generate the corresponding cyclo-Pro-AAs. For this purpose, linear-Val-Pro and linear-His-Pro were chosen as a model compound in the following experiments. The solutions of linear dipeptides with or without the presence of P_3_m were incubated at pH 11 and 55°C for 1 day. As expected, the corresponding cyclo-Pro-AA were detected from the resulting solutions by HPLC-HRMS (Supplementary Figure S15). As shown in the above results, the amount of cyclo-Pro-AA produced in the reaction system containing P_3_m was more than that without P_3_m (Supplementary Figure S16).

We next wondered if proline-containing cyclic tripeptide could be formed from corresponding linear-AA-AA-Pro mixture with P_3_m. For this purpose, we studied the reaction of linear-Ala-Gly-Pro and P_3_m. As expected, the cyclo-Pro-Gly-Ala was detected from the resulting solution by HPLC-HRMS (Supplementary Figure S17). It is worth noting that, although the yield of the cyclo-Pro-Gly-Ala was fairly low, this result was of significant meaning in the evolution process of life. Given the unfavorable transannular interactions and entropic factors, the synthesis of medium-sized rings (seven to nine membered rings) is challenging. In our case, cyclo-Pro-Gly-Ala is a nine-membered ring which is unfavorably compared to forming a small-sized ring (6 membered rings of cyclo-Pro-AA).

**SCHEME 1:**
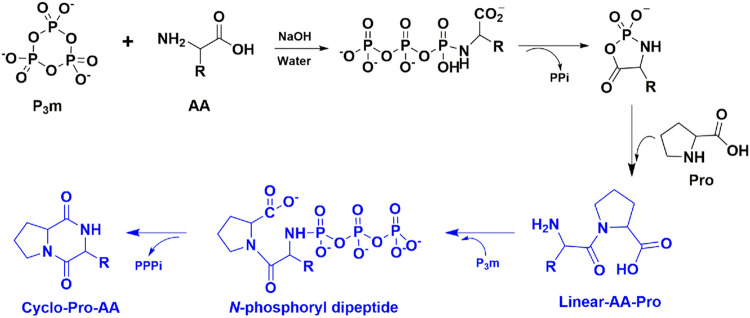
Mechanism for the cyclo-Pro-AAs formation, which may undergo the formation, and cyclization of linear dipeptide. This paper focuses on the blue part of the schema of the mechanism, that is, the linear dipeptide cyclization process. For the sake of convenience, we took the mechanism of cyclo-Pro-AA formation from linear-AA-Pro as an example. PPi and PPPi represent pyrophosphate and triphosphate, respectively.

Based on the above observation and taking into account the mechanism of cyclo-Pro-AA formation from amino acids, we could deduce a possible mechanism for the cyclo-Pro-AAs formation, which may undergo the formation and cyclization of linear dipeptide (Supplementary Figure S18). Since the mechanism for the linear dipeptide formation has been reported in the reaction of amino acids with P_3_m aqueous solution, we would not elaborate on this process (Chung et al., 1971; Inoue et al., 1993; Ni et al., 2009). In general, the proline and other amino acids react with P_3_m to form proline-containing linear dipeptide, then the linear dipeptide reacts with P_3_m to form N-phosphoryl dipeptide, Sequentially, N-phosphoryl dipeptide is hydrolyzed to release triphosphate (PPPi) and cyclized to cyclic dipeptide (Cyclo-Pro-AA) (Scheme 1).

## Conclusion

In summary, the proline-containing linear dipeptides (linear-Pro-AAs and linear-AAs-Pro) could spontaneously form the corresponding DKPs (cyclo-Pro-AAs) in the aqueous solution of pH 11. Importantly, the cyclo-Pro-AAs were more readily formed from linear-AA-Pro than linear-Pro-AA and the amount was promoted by P_3_m. The DKPs have the properties of peptide elongation and chiral catalysis, so they might play an important role in the chemical evolution of early life. These findings are very helpful for understanding the formation of DKPs in the prebiotic conditions and provide the possibility of prebiotic chemical reactions, such as peptide elongation and chiral catalysis, which occur without enzymes, but with a simpler small-molecule catalyst (DKPs).

## Data Availability

The original contributions presented in the study are included in the article/Supplementary Material, further inquiries can be directed to the corresponding authors.
