# Supplementary material for: Cyclic Dipeptides Formation From Linear Dipeptides Under Potentially Prebiotic Earth Conditions
Source: Front Chem. 2021 Jun 28;9:675821. doi: 10.3389/fchem.2021.675821 (PMC8273163; doi:10.3389/fchem.2021.675821)
Supplement: Supplementary file 1 [file DataSheet1.PDF]

# *Supplementary Material*

**Contents**

1 MS spectra analysis of reagent linear dipeptides and tripeptide ........................................2

2 Accurate mass measurements of cyclic dipeptides (Cyclo-Pro-AAs). ................................9

3 HPLC-MS spectra of cyclic dipeptides (Cyclo-Pro-AAs) ................................................10

4 HPLC-MS spectra of Cyclo-Pro-Gly-Ala .........................................................................19

5 Mechanism for the formation of Cyclo-Pro-AAs from amino acids. ................................20

6 Energy value for the linear dipeptide to form cyclic dipeptide ........................................21

# 1 MS spectra analysis of reagent linear dipeptides and tripeptide

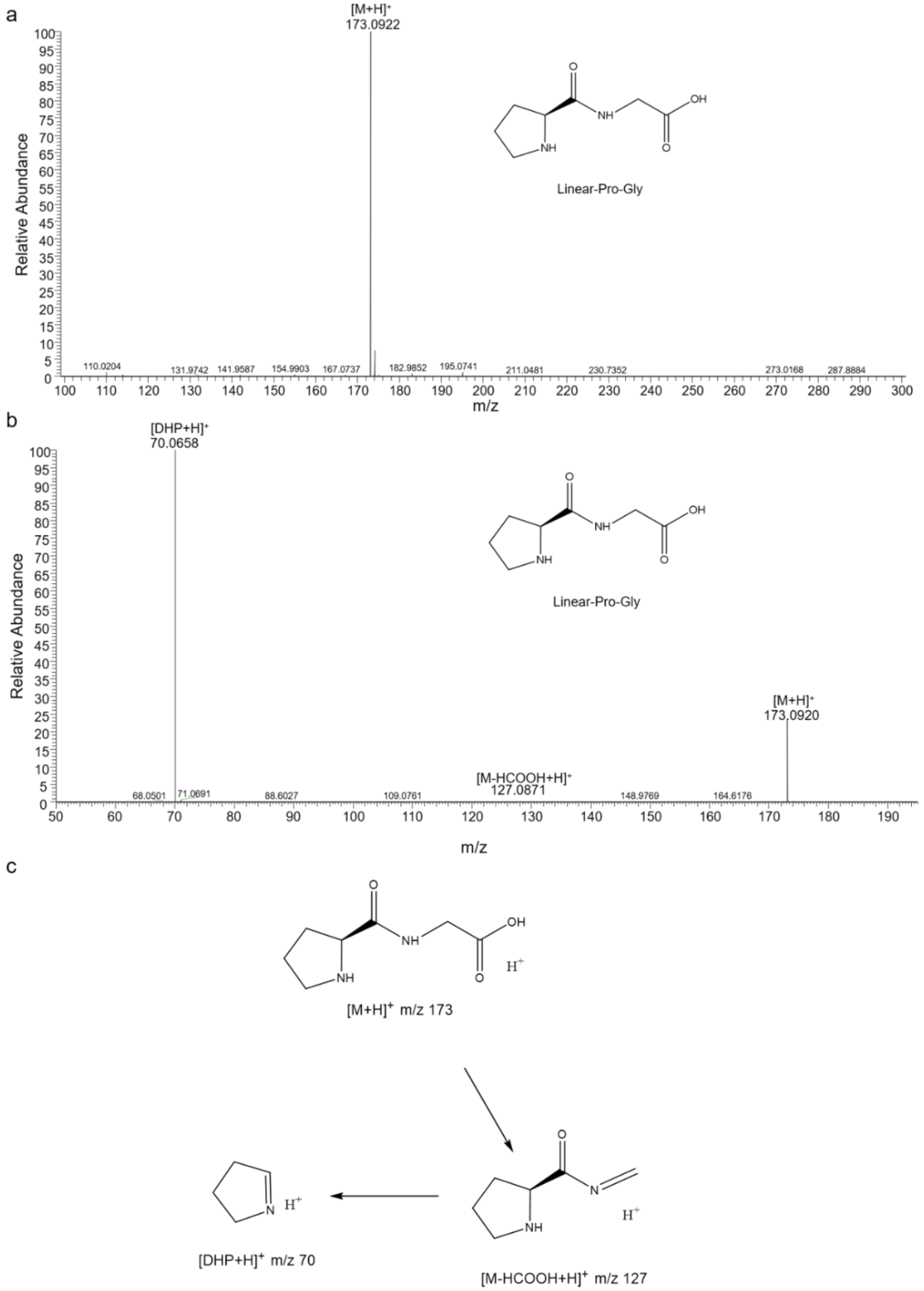


**Supplementary Figure 1** MS analysis of linear-Pro-Gly. a) The MS spectrum of linear-Pro-Gly (Mr=172). b) The $MS^2$ spectrum of linear-Pro-Gly. c) The $MS^2$ fragmentation pathways of linear-Pro-Gly. DHP: 3, 4-dihydro-2H-pyrrole.

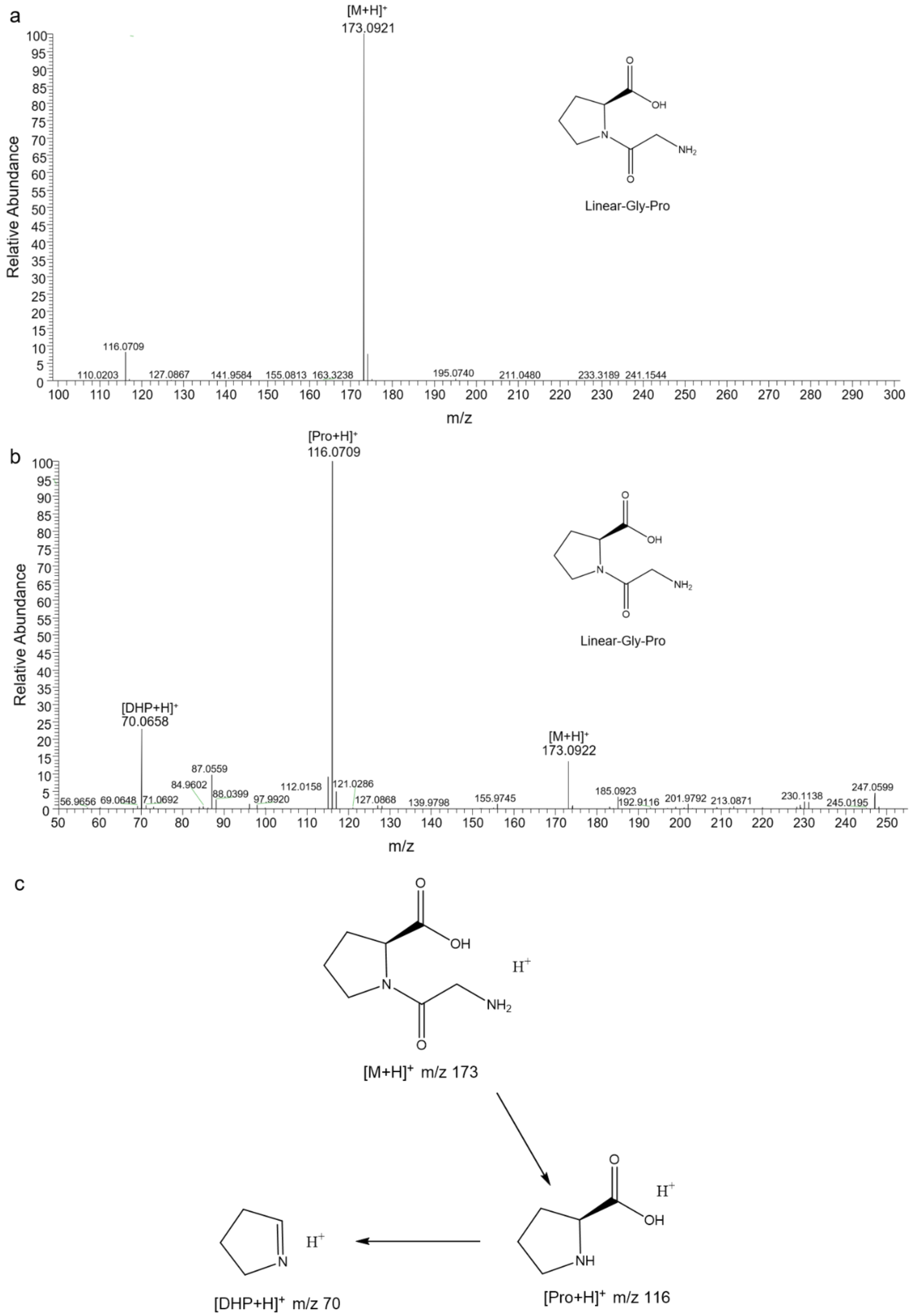


**Supplementary Figure 2** MS analysis of linear-Gly-Pro. a) The MS spectrum of linear-Gly-Pro (Mr=172). b) The $MS^2$ spectrum of linear-Gly-Pro. c) The $MS^2$ fragmentation pathways of linear-Gly-Pro. DHP: 3, 4-dihydro-2H-pyrrole.

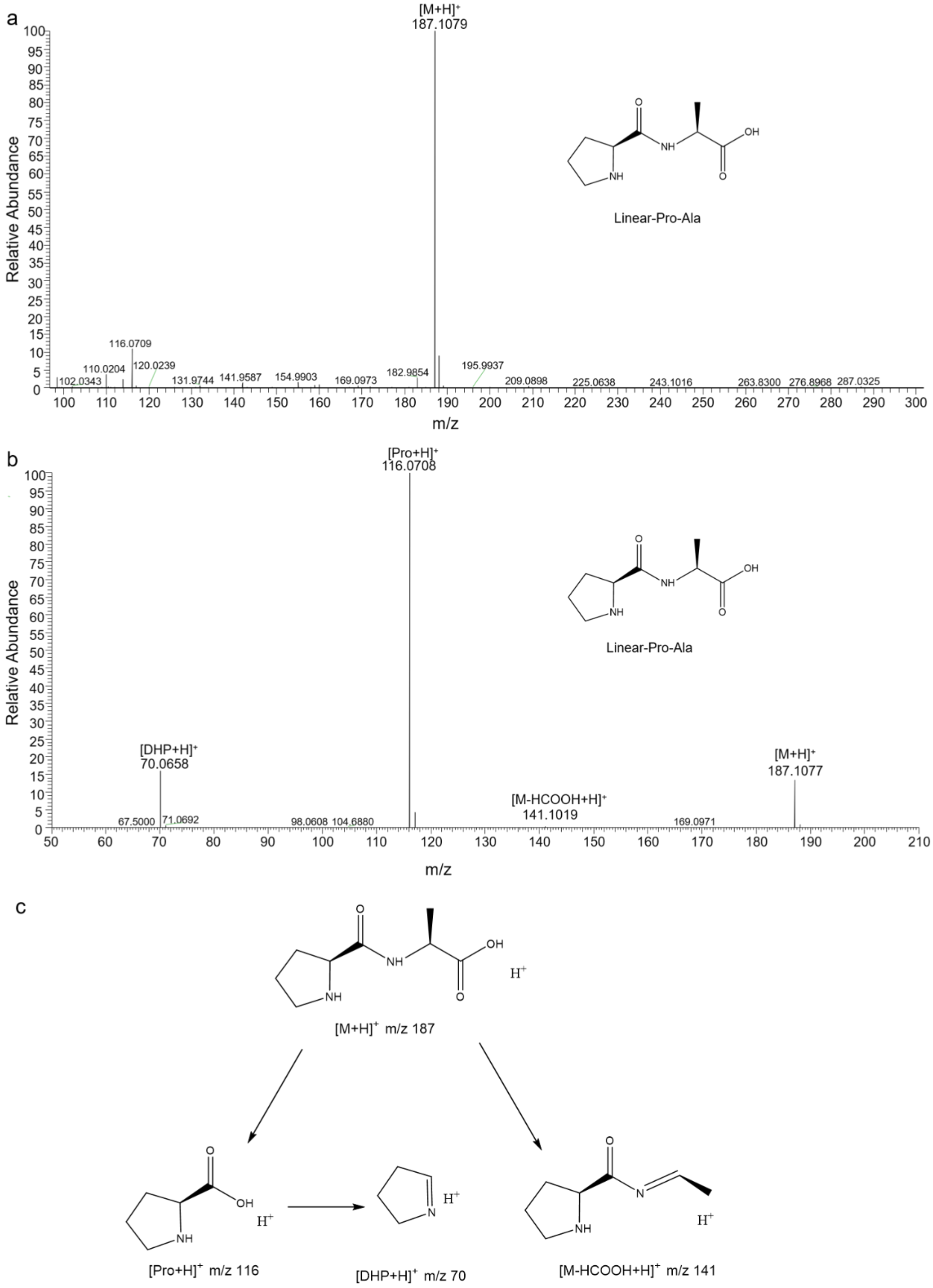


**Supplementary Figure 3** MS analysis of linear-Pro-Ala. a) The MS spectrum of linear-Pro-Ala (Mr=186). b) The MS$^2$ spectrum of linear-Pro-Ala. c) The MS$^2$ fragmentation pathways of linear-Pro-Ala. DHP: 3, 4-dihydro-2H-pyrrole.

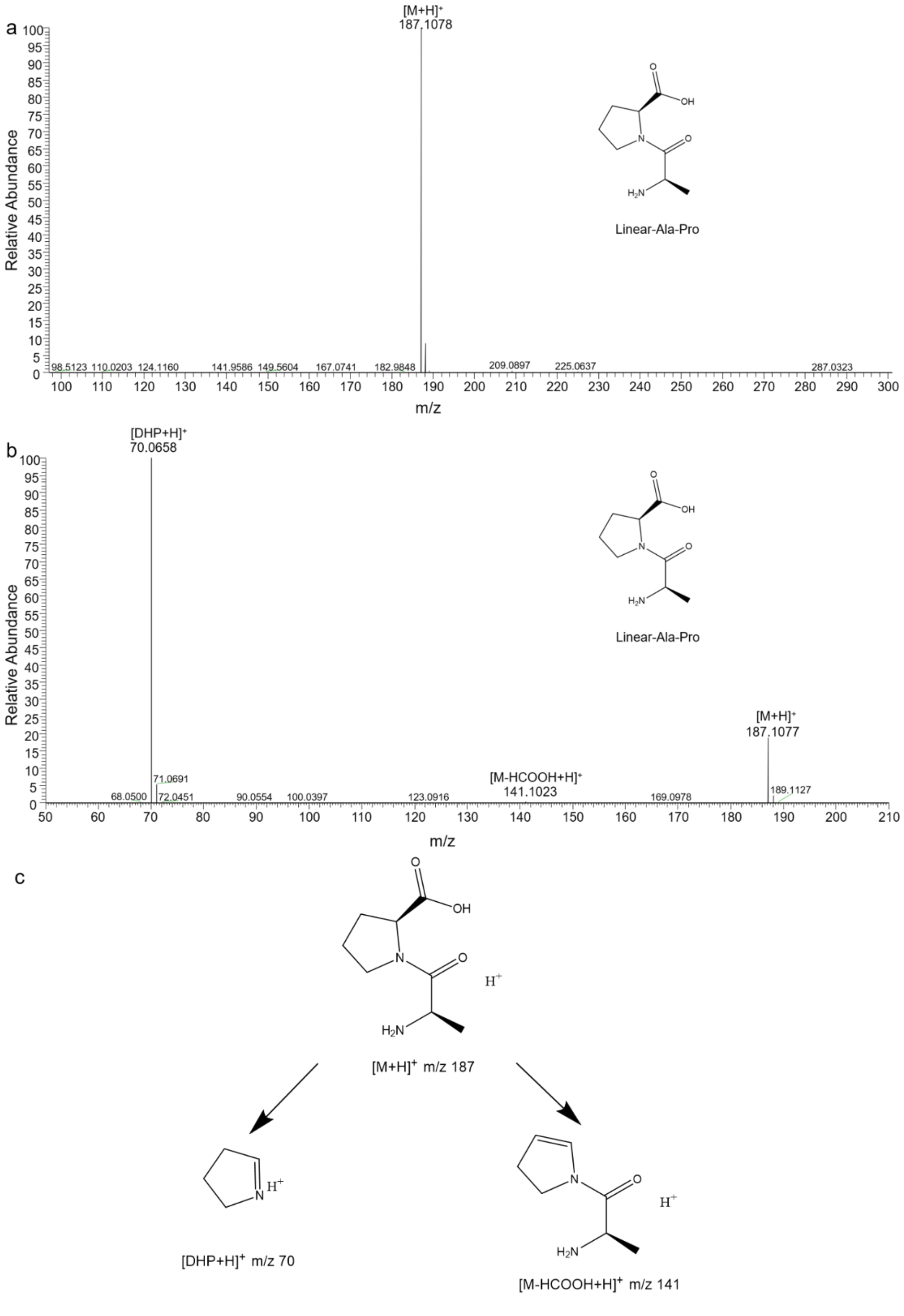


**Supplementary Figure 4** MS analysis of linear-Ala-Pro. a) The MS spectrum of linear-Ala-Pro (Mr=186). b) The $MS^2$ spectrum of linear-Ala-Pro. c) The $MS^2$ fragmentation pathways of linear-Ala-Pro. DHP: 3, 4-dihydro-2H-pyrrole.

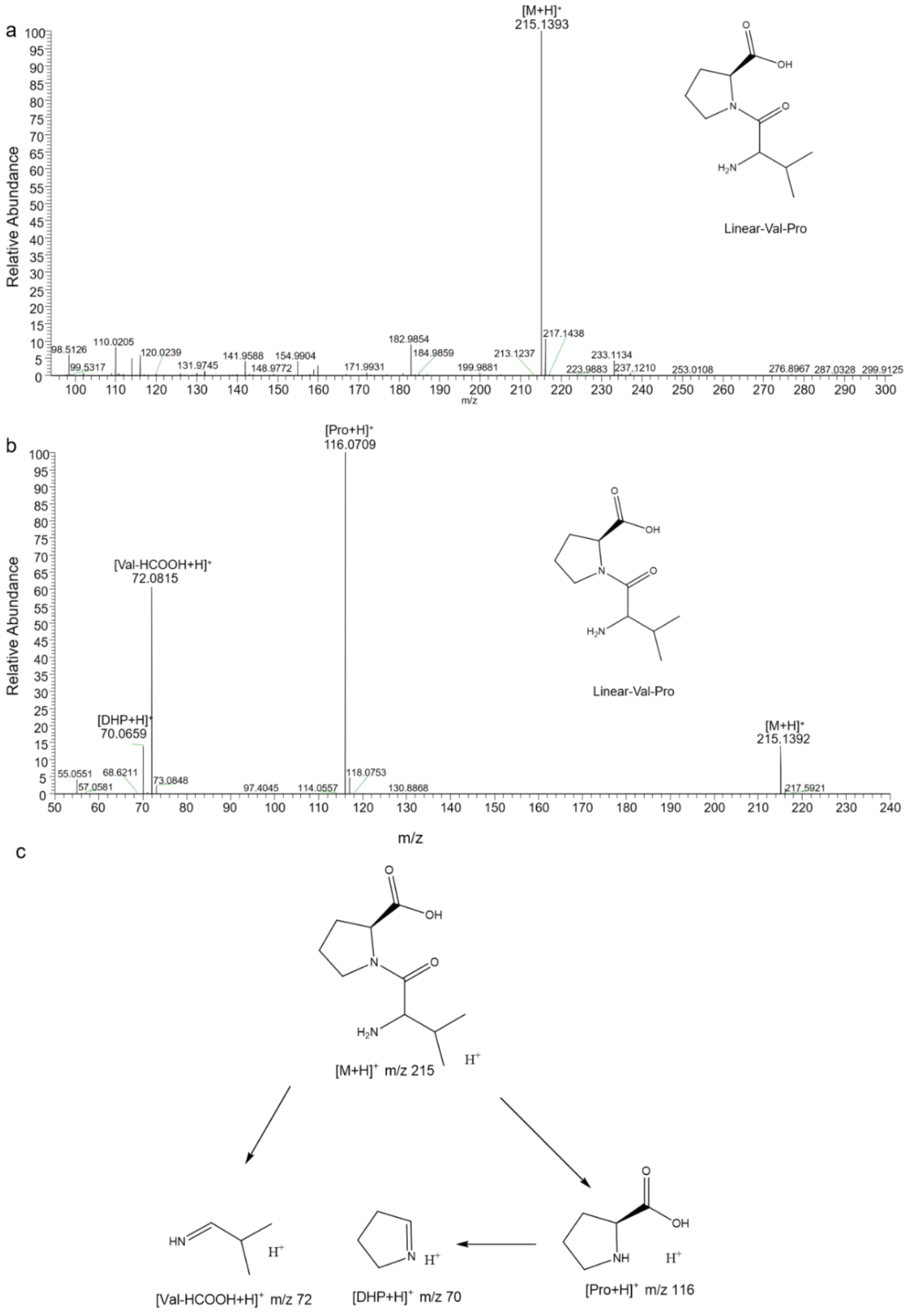


**Supplementary Figure 5** MS analysis of linear-Val-Pro. a) The MS spectrum of linear-Val-Pro (Mr=214). b) The $MS^2$ spectrum of linear-Val-Pro. c) The $MS^2$ fragmentation pathways of linear-Val-Pro. DHP: 3, 4-dihydro-2H-pyrrole.

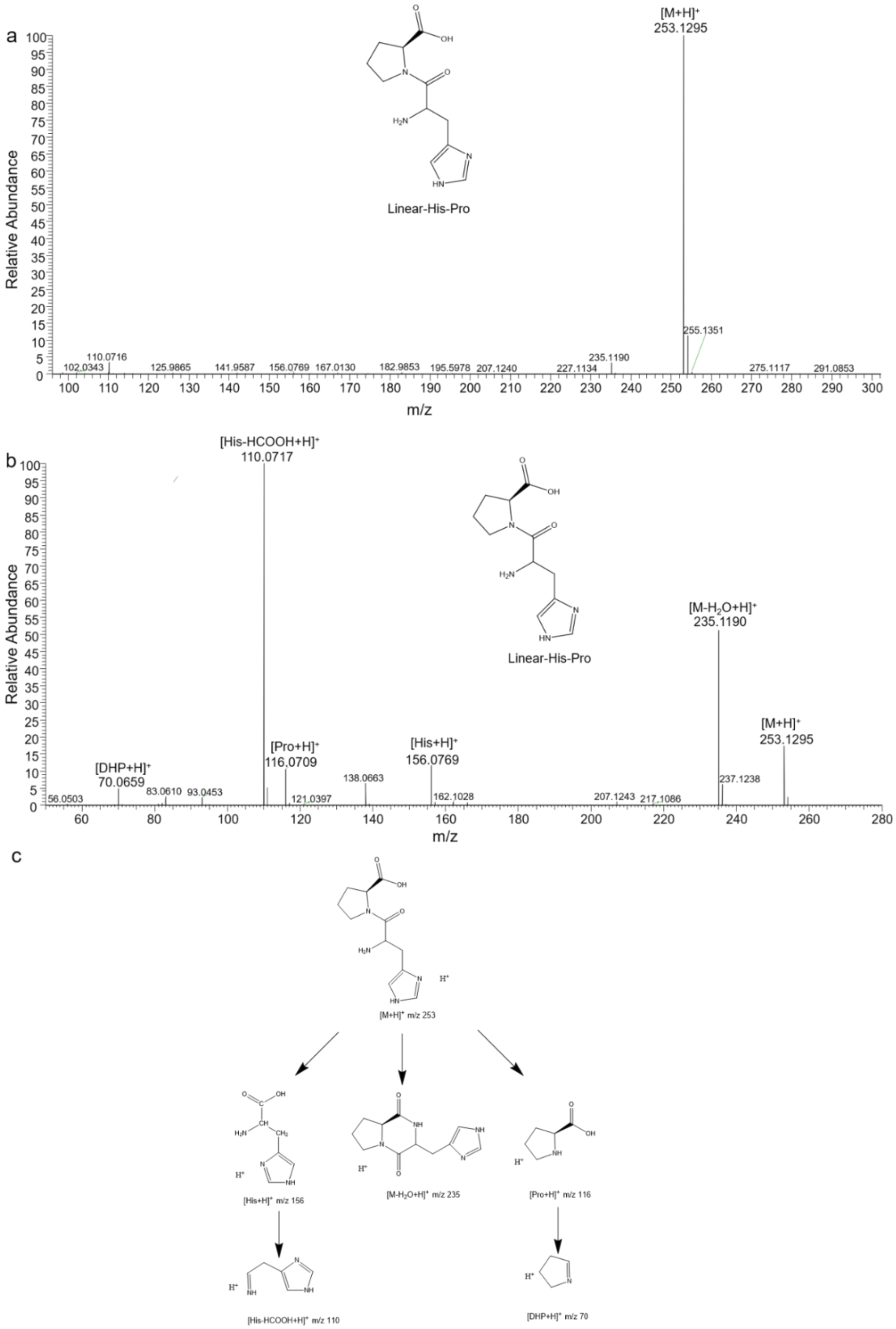


**Supplementary Figure 6** MS analysis of linear-His-Pro. a) The MS spectrum of linear-His-Pro (Mr=252). b) The $MS^2$ spectrum of linear-His-Pro. c) The $MS^2$ fragmentation pathways of linear-His-Pro. DHP: 3, 4-dihydro-2H-pyrrole.

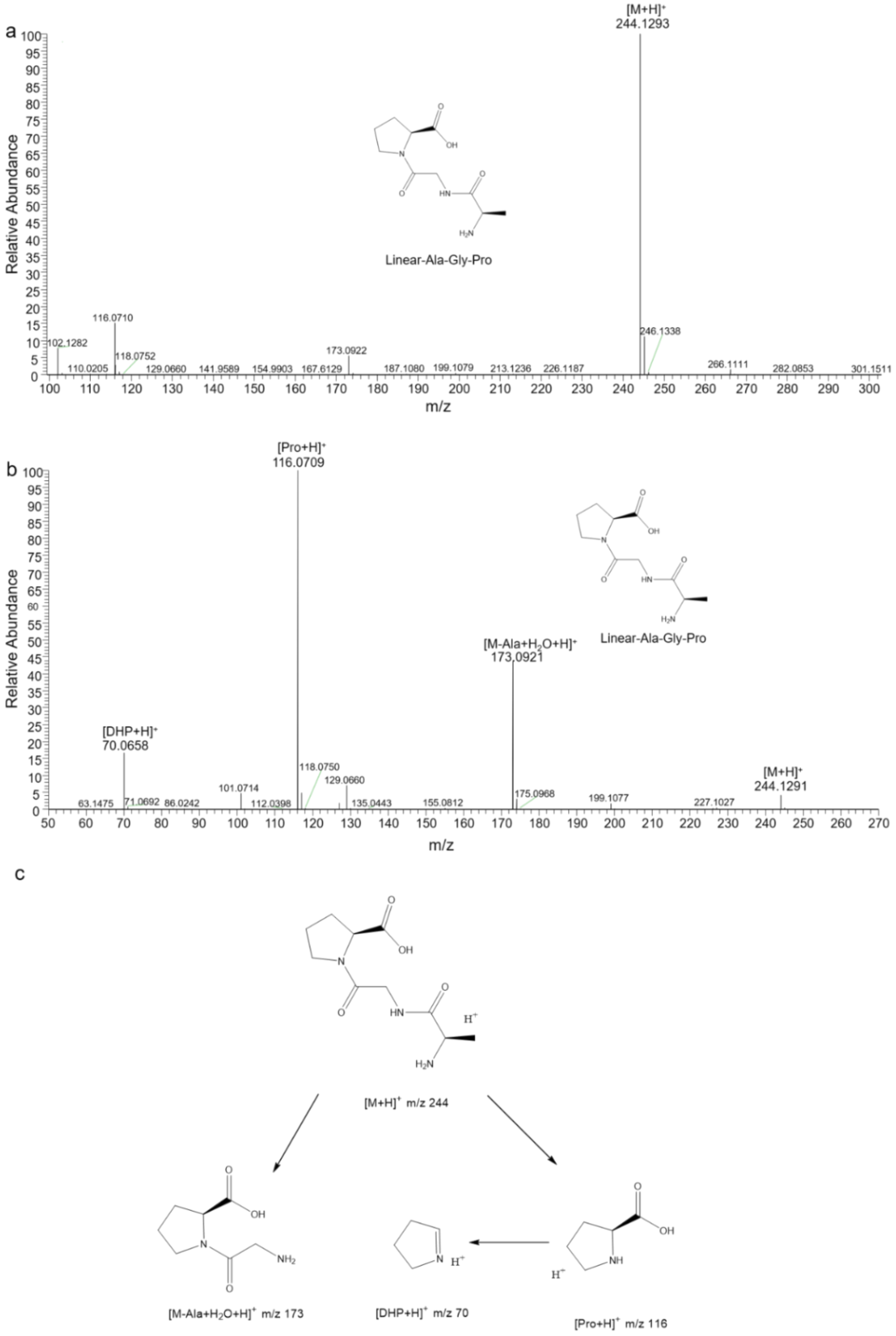


**Supplementary Figure 7** MS analysis of linear-Ala-Gly-Pro. a) The MS spectrum of linear-Ala-Gly-Pro (Mr=243). b) The $MS^2$ spectrum of linear-Ala-Gly-Pro. c) The $MS^2$ fragmentation pathways of linear-Ala-Gly-Pro. DHP: 3, 4-dihydro-2H-pyrrole.

## 2 Accurate mass measurements of cyclic dipeptides (Cyclo-Pro-AAs).

**Supplementary Table 1** Accurate mass measurements of cyclo-Pro-AAs

| Reactant | Product / *m / z* | Cyclo-Pro-Gly | Cyclo-Pro-Ala | Cyclo-Pro-Val | Cyclo-Pro-His | Cyclo-Pro-Gly-Ala |
|---|---|---|---|---|---|---|
| | | $[M+H]^+$ | $[M+H]^+$ | $[M+H]^+$ | $[M+H]^+$ | $[M+H]^+$ |
| Linear-Pro-Gly | *Cal .*[a] | 155.0815 | | | | |
| | *Exp.* [b] | 155.0816 | | | | |
| | *Δ ppm* [c] | 0.6 | | | | |
| Linear-Gly-Pro | *Cal .*[a] | 155.0815 | | | | |
| | *Exp.* [b] | 155.0814 | | | | |
| | *Δ ppm* [c] | 0.6 | | | | |
| Linear-Pro-Ala | *Cal .*[a] | | 169.0972 | | | |
| | *Exp.* [b] | | 169.0974 | | | |
| | *Δ ppm* [c] | | 1.2 | | | |
| Linear-Ala-Pro | *Cal .*[a] | | 169.0972 | | | |
| | *Exp.* [b] | | 169.0987 | | | |
| | *Δ ppm* [c] | | 8.9 | | | |
| Linear-Val-Pro | *Cal .*[a] | | | 197.1285 | | |
| | *Exp.* [b] | | | 197.1285 | | |
| | *Δ ppm* [c] | | | 0 | | |
| Linear-His-Pro | *Cal .*[a] | | | | 235.1190 | |
| | *Exp.* [b] | | | | 235.1196 | |
| | *Δ ppm* [c] | | | | 2.6 | |
| Linear-Ala-Gly-Pro | *Cal .*[a] | | | | | 226.1186 |
| | *Exp.* [b] | | | | | 226.1185 |
| | *Δ ppm* [c] | | | | | 0.4 |

[a] *Cal.*: The calculated value of the compound $[M+H]^+$. [b] *Exp.*: The experimental value the compound $[M+H]^+$.

[c] *Δ ppm*: The relative error between the calculated value and the experimental value. Most of *Δ ppm* are less than 5 ppm.

The experimental values shown in the above table are all measured in the presence of $P_3m$ in the reaction solution.

### 3 HPLC-MS spectra of cyclic dipeptides (Cyclo-Pro-AAs)

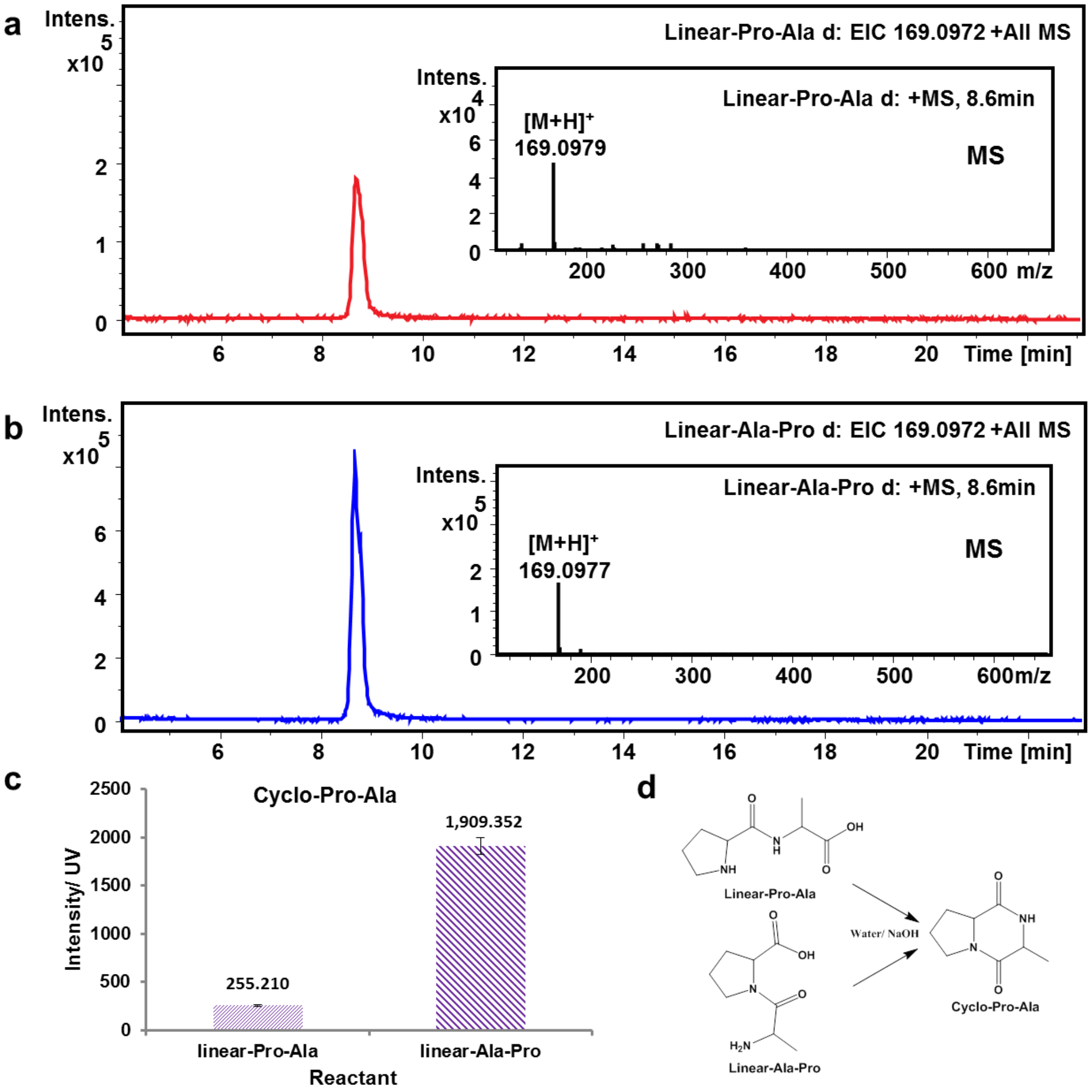


**Supplementary Figure 8** Formation of cyclo-Pro-Ala from the cyclization of linear dipeptide in an alkaline aqueous solution (pH= 11). The calculated value of cyclo-Pro-Ala $[M+H]^+$ is 169.0972. (a) EIC-MS profile of the product cyclo-Pro-Ala from the cyclization of linear-Pro-Ala. (b) EIC-MS profile of the product cyclo-Pro-Ala from the cyclization of linear-Ala-Pro. (c) The amount of cyclo-Pro-Ala were produced from linear-Pro-Ala and linear-Gly-Ala, respectively. (d) The reaction of the cyclo-Pro-Ala formation through the cyclization of linear-Pro-Ala and linear-Ala-Pro, respectively.

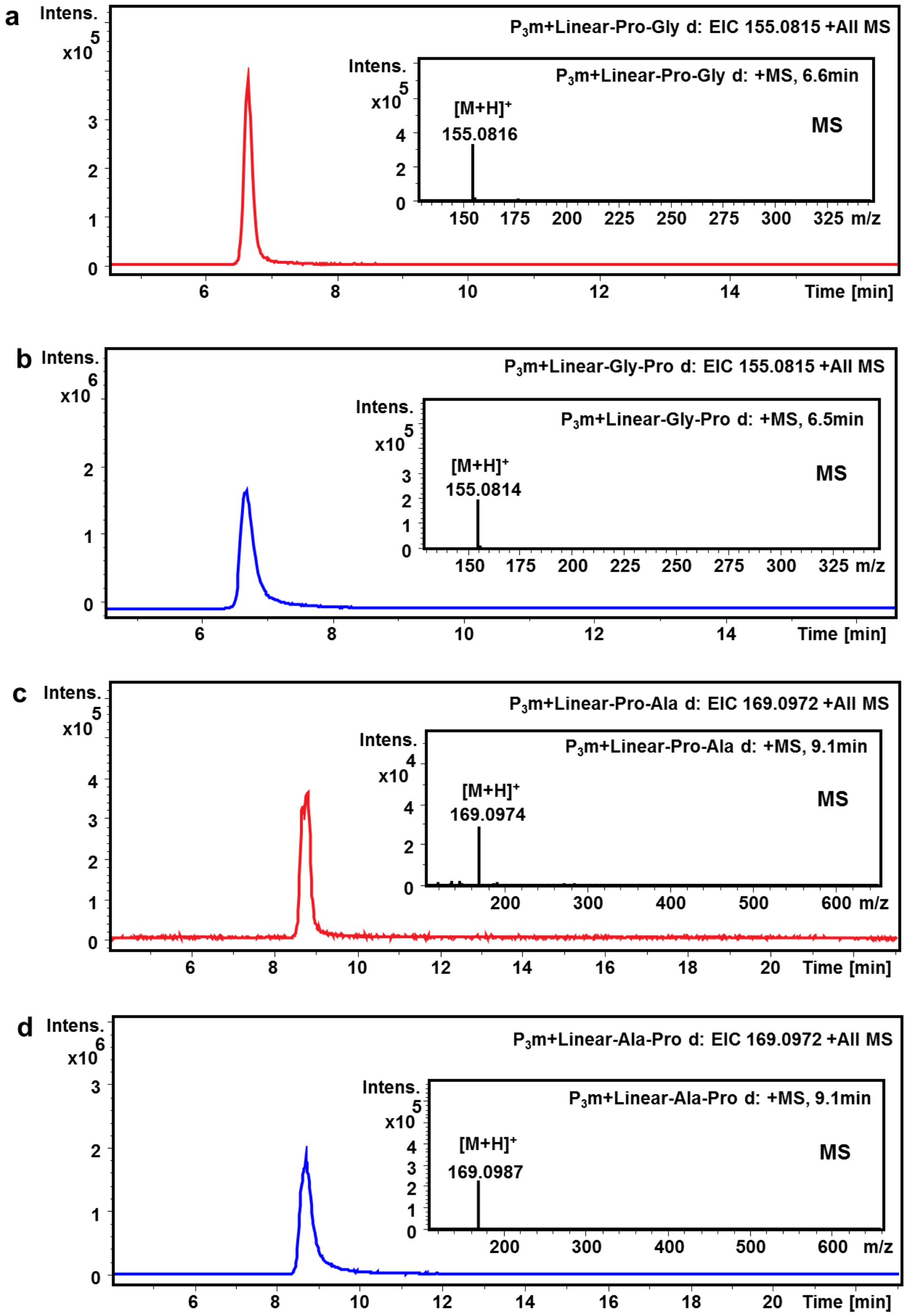


**Supplementary Figure 9** HPLC-MS-EIC spectra for the product cyclic dipeptides of linear dipeptides with trimetaphosphate ($P_3m$). The EIC spectra of $[M+H]^+$ give 2 peaks, which indicated that the cyclic dipeptides have two isomers and it might be that the polarity differences of some isomers are too small so that their peaks are overlapped. (a) reaction of linear-Pro-Gly with $P_3m$. (b) reaction of linear-Gly-Pro with $P_3m$. (c) reaction of linear-Pro-Ala with $P_3m$. (d) reaction of linear-Ala-Pro with $P_3m$.

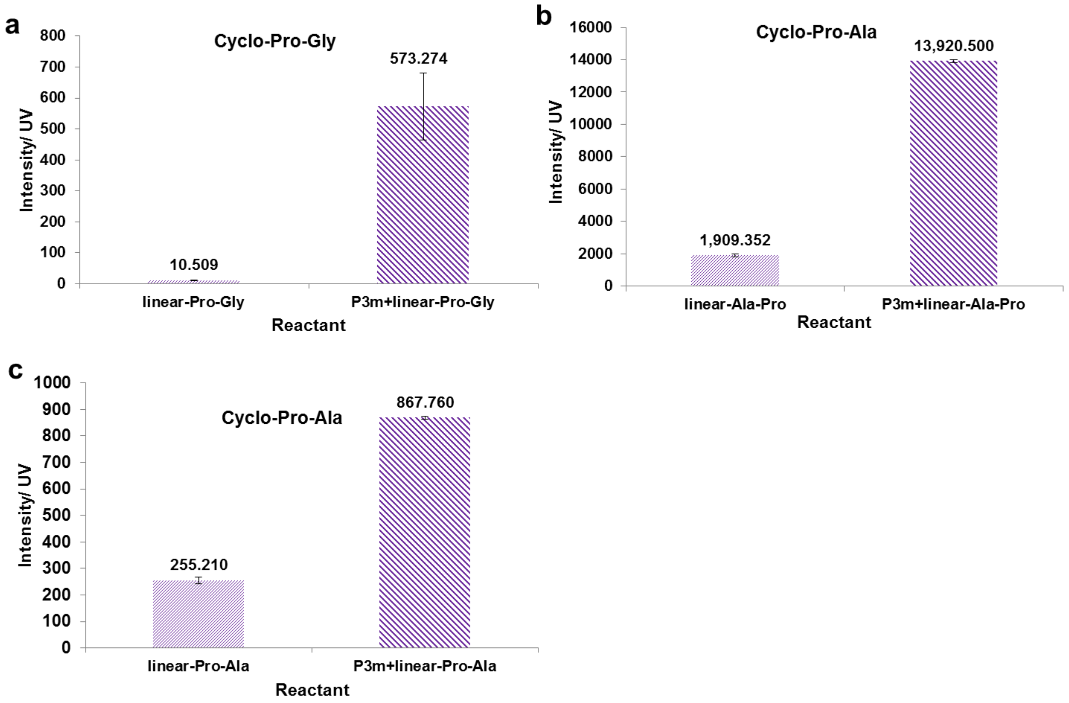


**Supplementary Figure 10** Effects of $P_3m$ on the formation of cyclo-Pro-AAs in the reaction of linear dipeptides with or without the presence of $P_3m$. (a) the reaction of linear-Pro-Gly with or without the presence of $P_3m$. (b) the reaction of linear-Ala-Pro with or without the presence of $P_3m$. (c) the reaction of linear-Pro-Ala with or without the presence of $P_3m$. All reactions were controlled under standard conditions.

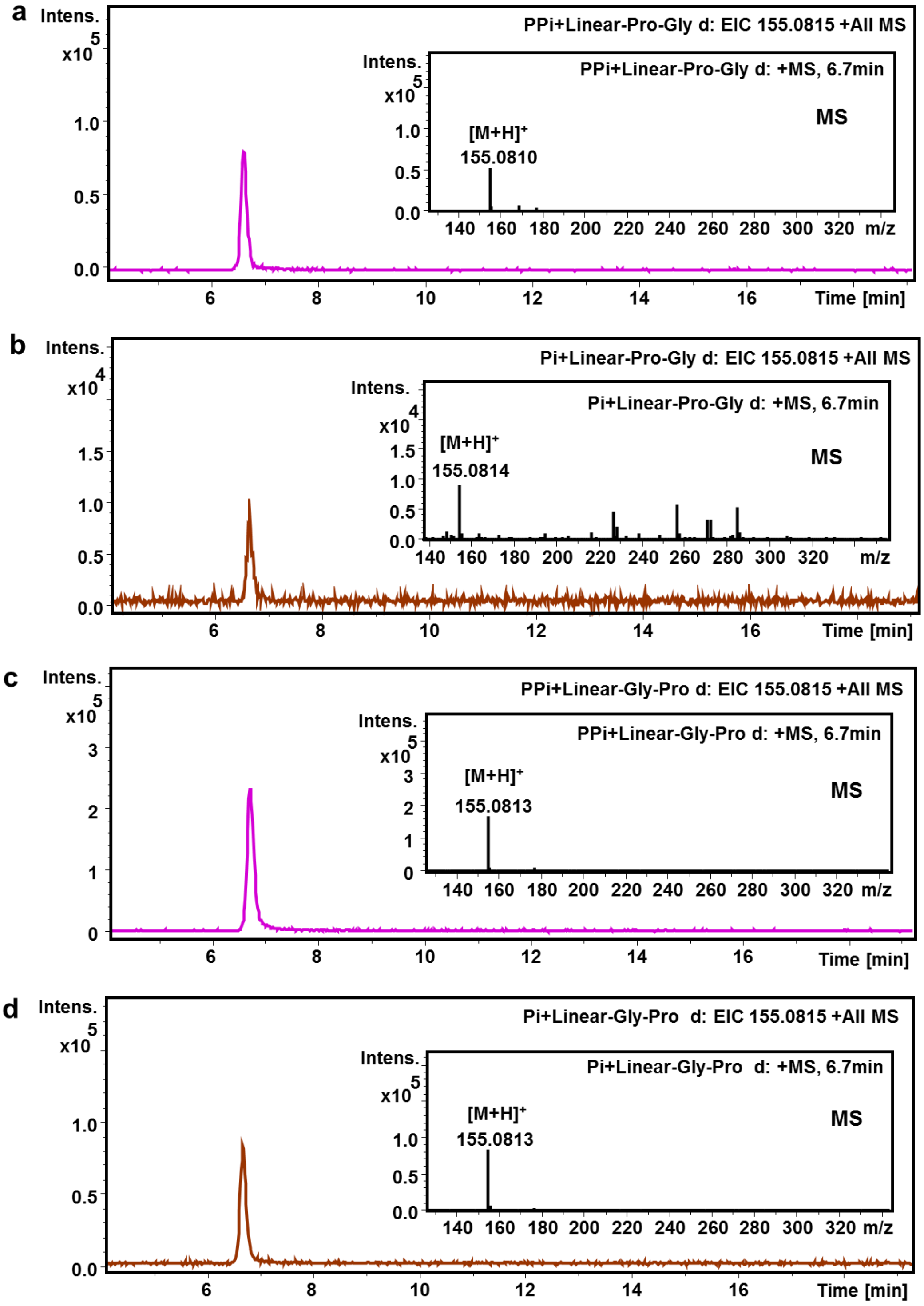


**Supplementary Figure 11** HPLC-MS-EIC spectra for the product cyclo-Pro-Gly of linear dipeptides with different salt. (a) the reaction of linear-Pro-Gly with PPi. (b) the reaction of linear-Pro-Gly with Pi. (c) the reaction of linear-Gly-Pro with PPi. (d) the reaction of linear-Gly-Pro with Pi.

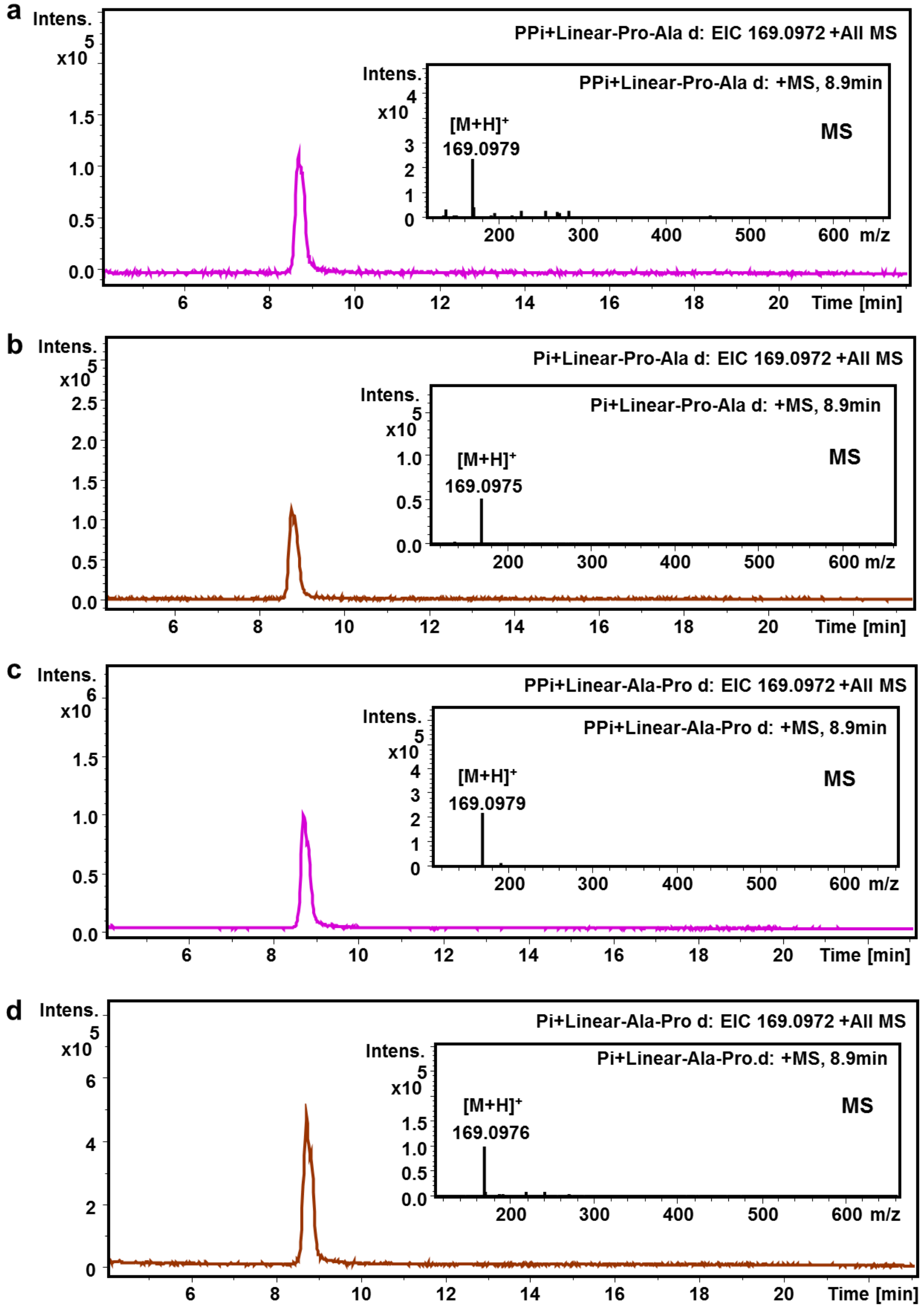


**Supplementary Figure 12** HPLC-MS-EIC spectra for the product cyclo-Pro-Ala of linear dipeptides with different salt. (a) the reaction of linear-Pro-Ala with PPi. (b) the reaction of linear-Pro-Ala with Pi. (c) the reaction of linear-Ala-Pro with PPi. (d) the reaction of linear-Ala-Pro with Pi.

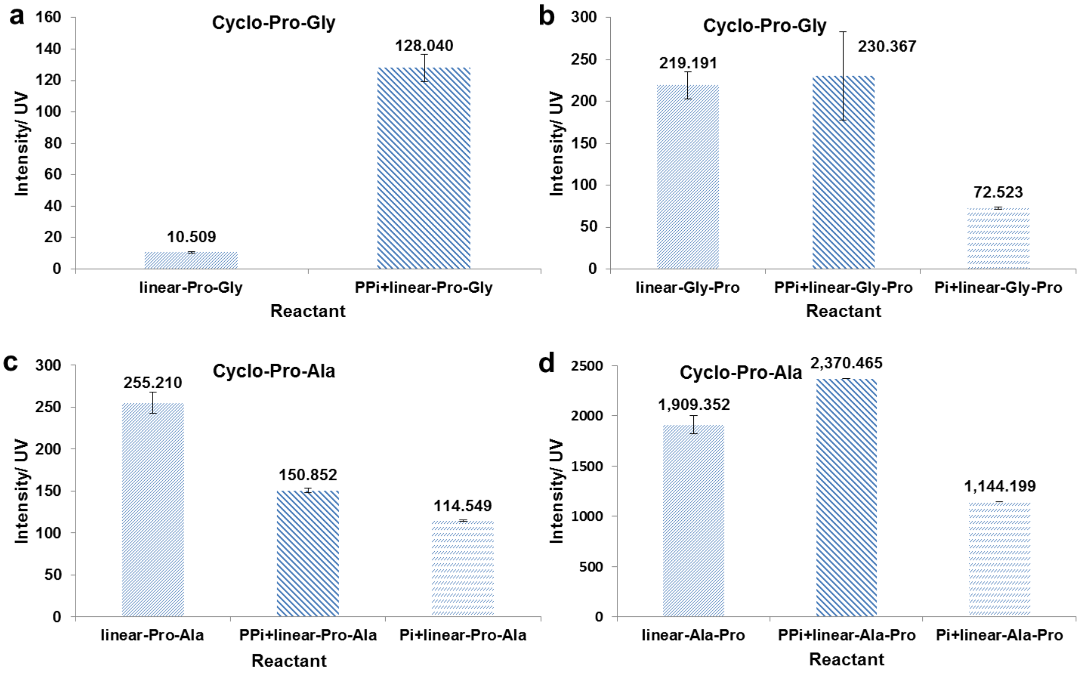


**Supplementary Figure 13** Effects of PPi and Pi on the formation of cyclo-Pro-AAs. (a) the reaction of linear-Pro-Gly with or without the presence of PPi. It is worth noting that the amount of cyclo-Pro-Gly generated by linear-Pro-Gly reaction with Pi was too small to be quantized, so its experimental results were not shown. (b) the reaction of linear-Gly-Pro with or without the presence of PPi and Pi, respectively. (c) the reaction of linear-Pro-Ala with or without the presence of PPi and Pi, respectively. (d) the reaction of linear-Ala-Pro with or without the presence of PPi and Pi, respectively. PPi: Pyrophosphate; Pi: Phosphate. All reactions were controlled under standard conditions. These results showed that PPi and Pi have not all positive effect on the yields of cyclo-Pro-AAs.

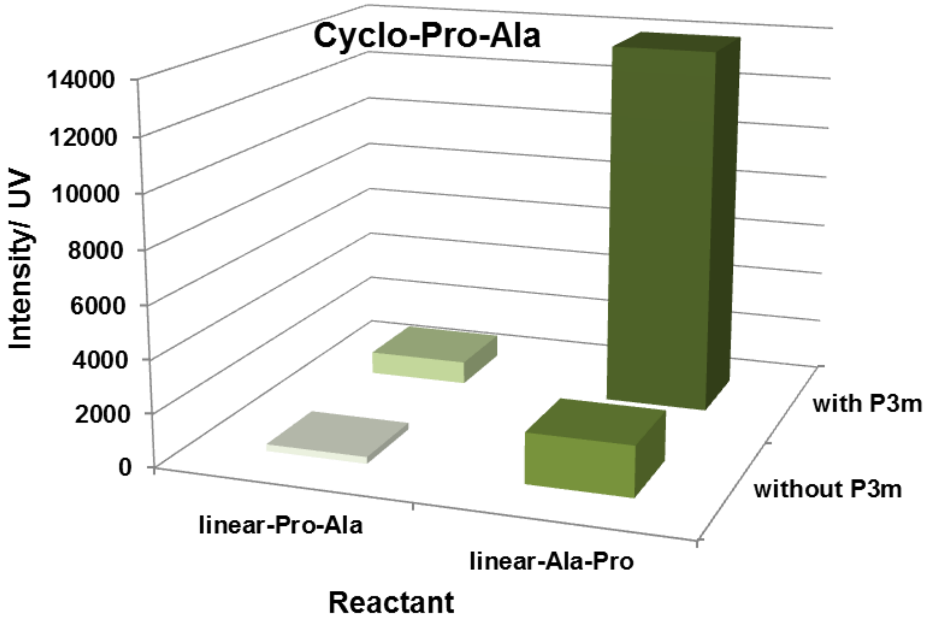


**Supplementary Figure 14** Effect of $P_3m$ and the sequence of linear dipeptides on the formation of cyclo-Pro-Ala. All reactions were controlled under standard conditions.

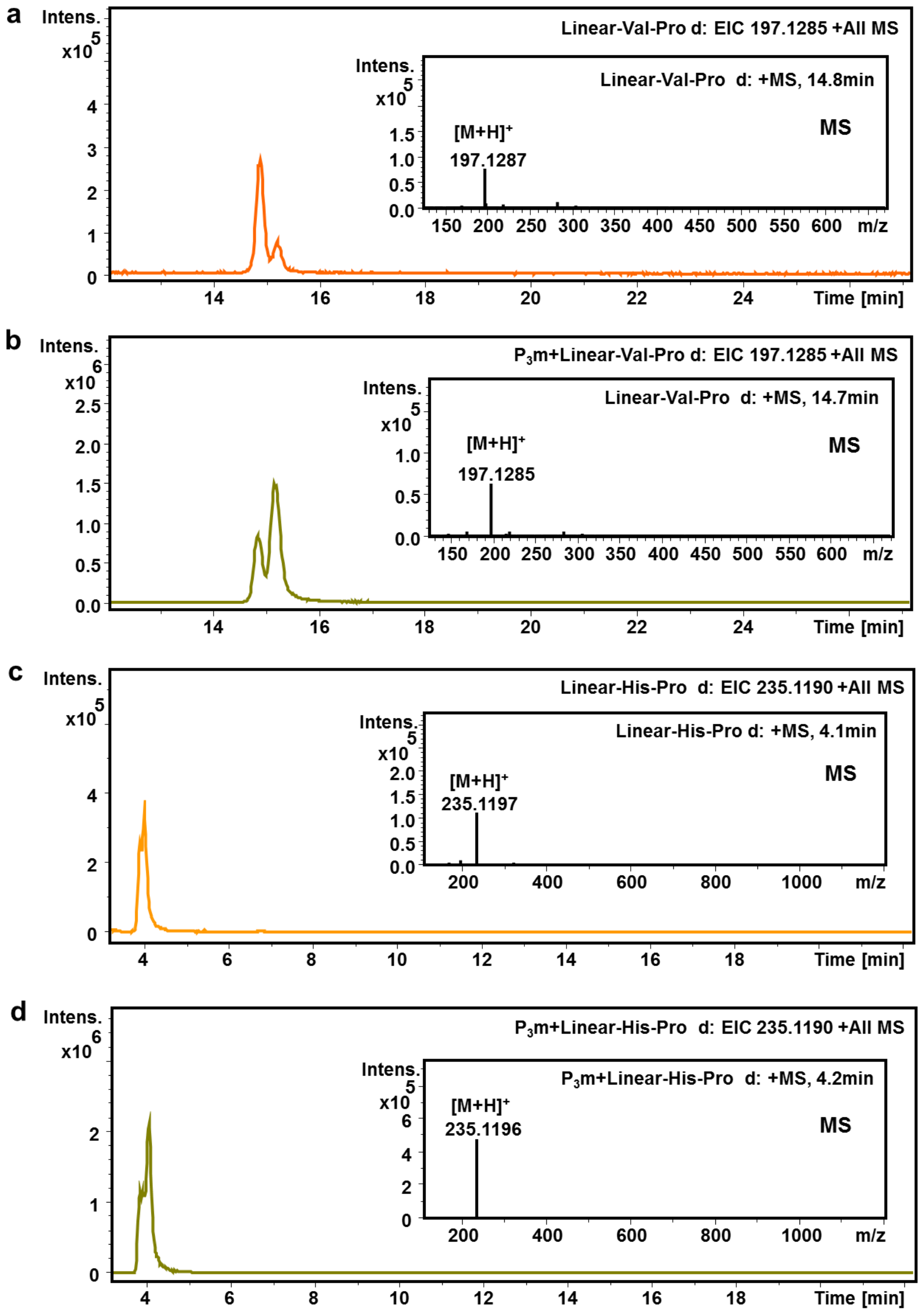


**Supplementary Figure 15** HPLC-MS-EIC spectra for the product cyclo-Pro-AAs of linear dipeptides with or without $P_3m$. The EIC spectra of $[M+H]^+$ give 2 peaks, which indicated that the cyclo-Pro-AAs have two isomers and it might be that the polarity differences of some isomers are too small so that their peaks are overlapped. (a) formation of cyclo-Pro-Val from the cyclization of the linear-Val-Pro. (b) formation of cyclo-Pro-Val from the cyclization of the linear-Val-Pro with the presence of the $P_3m$. (c) formation of cyclo-Pro-His from the cyclization of the linear-His-Pro. (d) formation of cyclo-Pro-His from the cyclization of the linear-His-Pro with the presence of the $P_3m$.

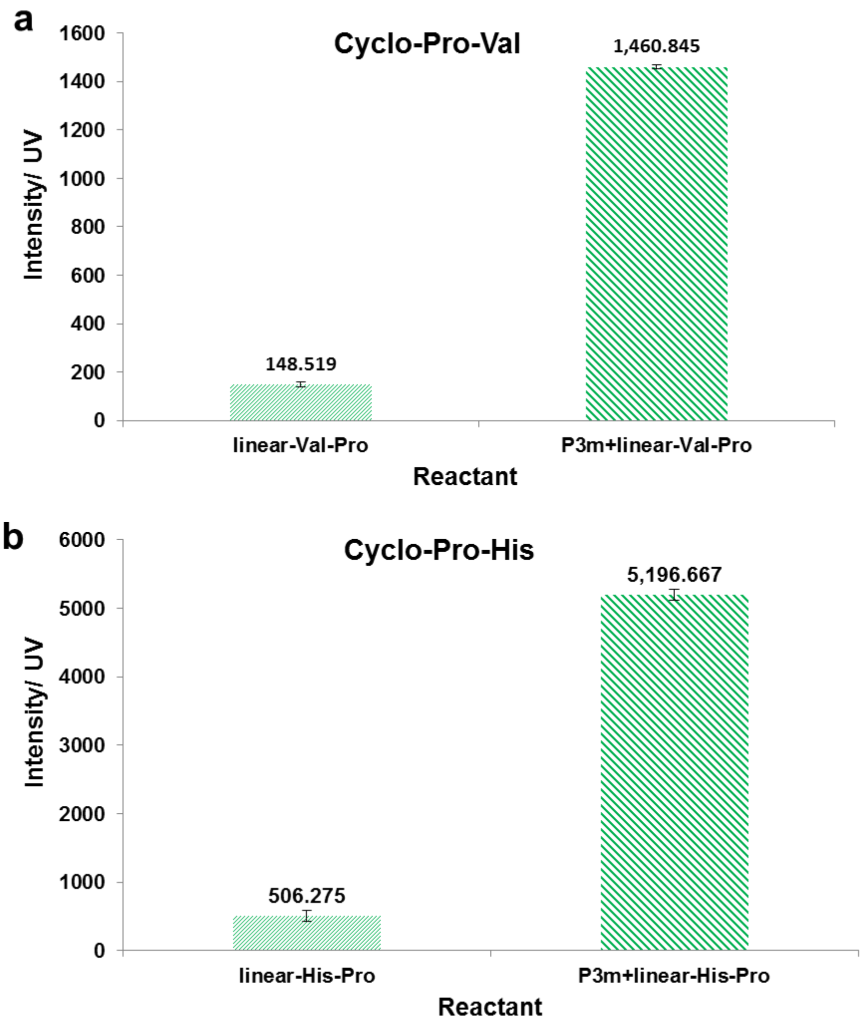


**Supplementary Figure 16** Effects of $P_3m$ on the formation of cyclo-Pro-AAs in the reaction of linear dipeptides with or without the presence of $P_3m$. (a) the reaction of linear-Val-Pro with or without the presence of $P_3m$. (b) the reaction of linear-His-Pro with or without the presence of $P_3m$. All reactions were controlled under standard conditions.

## 4 HPLC-MS spectra of Cyclo-Pro-Gly-Ala

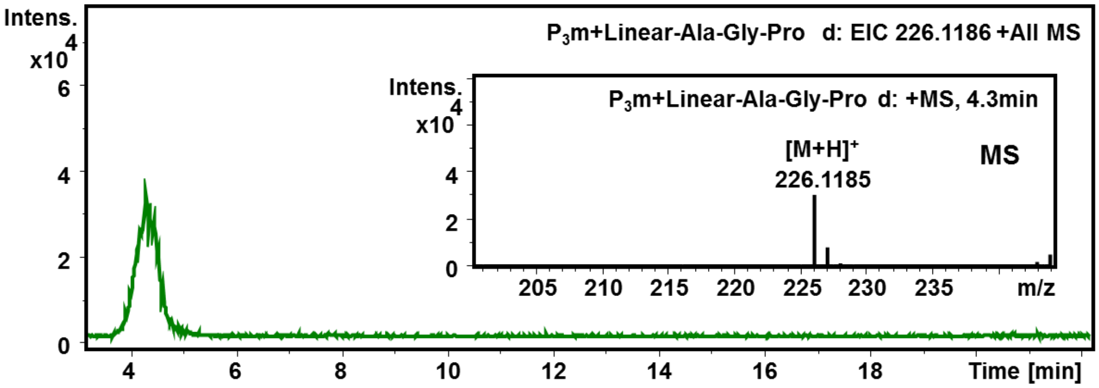


**Figure S17** HPLC-MS-EIC spectra for the product cyclo-Pro-Gly-Ala of linear-Ala-Gly-Pro with $P_3m$

## 5 Mechanism for the formation of Cyclo-Pro-AAs from amino acids.

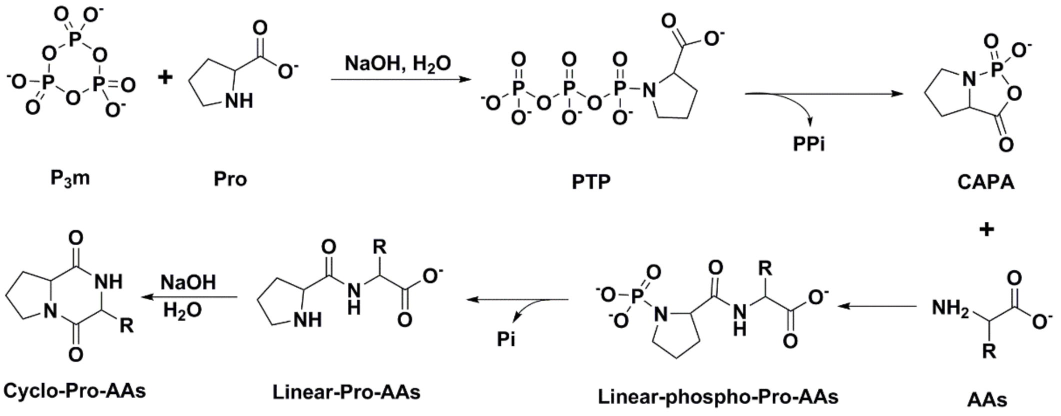


**Supplementary Figure 18** A possible mechanism for the formation of proline-containing cyclic dipeptides from amino acids.

PTP: Proline triphosphate; CAPA: Cyclic acylphosphoramidates

## 6 Energy value for the linear dipeptide to form cyclic dipeptide

**Supplementary Table 2** Energy value for the Linear-Gly-Pro and Linear-Pro-Gly to form cyclo-Pro-Gly

| | OPT/B1 | | OPT/B1+ZPE | | G | | H | |
|---|---|---|---|---|---|---|---|---|
| RC-LPG | -609.238518 | 1.4 | -609.038252 | 1.1 | -609.078615 | -0.2 | -609.025544 | 1.1 |
| RC-LGP | -609.240770 | 0.0 | -609.040053 | 0.0 | -609.078375 | 0.0 | -609.027347 | 0.0 |
| TS-LGP | -609.184581 | 35.3 | -608.987200 | 33.2 | -609.022157 | 35.3 | -608.976055 | 32.2 |
| PC-LGP | -609.251866 | -7.0 | -609.052671 | -7.9 | -609.091377 | -8.2 | -609.039506 | -7.6 |

Absolute energies are in au units and the relative ones are in kcal·mol$^{-1}$ units. LPG: Linear-Pro-Gly; LGP: Linear-Gly-Pro.
